# Naringin mitigated doxorubicin-induced kidney injury by the reduction of oxidative stress and inflammation with a synergistic anticancer effect

**DOI:** 10.1186/s40360-025-00947-7

**Published:** 2025-06-23

**Authors:** Nahla S. Gad, Sameh M. Shabana, Maggie E. Amer, Azza I. Othman, Mohamed A. El-Missiry

**Affiliations:** Zoology Department, Faculty of Science, Mansoura University, Mansoura, 35516 Egypt

**Keywords:** Naringin, Doxorubicin, Kidney injury, Antioxidants, Inflammation, Kidney histopathology

## Abstract

**Background:**

The pathophysiology and severity of kidney impairment due to doxorubicin (DOX) treatment are markedly influenced by oxidative stress and inflammation. Naringin (NG), a natural flavonoid, has anti-inflammatory and antioxidant properties. The nephroprotective effect of NG on DOX-induced kidney toxicity was investigated to increase its utility in clinical settings.

**Methods:**

DOX toxicity was induced by a single ip injection (15 mg/kg) and for possible protection NG (100 mg/Kg) was used.

**Results:**

Kidney damage and dysfunction were indicated by an elevation in the levels of creatinine, urea, uric acid, and the activity of ALP and LDH in serum, KIM-1, and NAGAL in kidney, and a significant decrease in nephrin and podocin in renal tissue. These disrupted glomerular and tubular function indicators were remarkably ameliorated by oral administration of NG (100 mg/kg) daily for 10 days before DOX treatment and continued for an additional four days post-Dox treatment. The nephroprotective effect of NG was confirmed by the improvement of histopathological and PAS histochemical investigations. The mitigating impact of NG was verified by normalization of the redox balance, evidenced by a significant amelioration of ROS levels, oxidative stress markers (MDA, PC, 8-OHdG), and antioxidants (GSH, GPx, GR), as well as upregulation of Nrf2 expression in kidney. Furthermore, NG significantly prevented the increase in the inflammatory mediators (IL-6, IL-1β, TNF-α, and NF-κB) and upregulated the anti-inflammatory IL-10 in DOX-treated rats. The expression of TGF-β1 and the apoptotic protein caspase-3 in the kidneys significantly decreased as a result of the improvement in redox state in renal tissue. Additionally, NG demonstrated anticancer effects and their combination showed synergistic anticancer impact on larynx and colon cancer cell lines *in vitro* study.

**Conclusions:**

NG demonstrated remarkable protection of kidney against DOX treatment.

## Background

Doxorubicin (DOX) is an antibiotic that exhibits remarkable chemotherapeutic efficacy against a variety of malignancies in both adult and pediatric patients. Kidney impairment caused by DOX presents a major challenge to cancer treatment. About 60% of cancer patients undergoing chemotherapy suffer nephrotoxicity, which limits its therapeutic effectiveness [1]. Nephrotoxicity has a significant risk of morbidity and death due to renal dysfunction which includes reduced filtration, reabsorption, and excretion [2]. The overproduction of reactive oxygen species (ROS) and the development of oxidative stress are the probable mechanisms [3]

Among others, renal tubular cytotoxicity, glomerulus and interstitial nephritis are the main kidney dysfunction induced by DOX. Podocytes are specialized epithelial cells that cover the glomerular basement membrane and regulate filtration process. Podocyte injury is one of the main characteristics of glomerular disorders in nephrotoxicity. Dox provoked marked podocyte apoptosis and severe proteinuria [4]. Nephrin and podocin are podocyte associated proteins that are essential for maintaining the structure integrity of slit diaphragm structural integrity [5]. Reduced nephrin and podocin expressions were observed in proteinuria glomerular diseases [6]. In a dose-dependent manner, Dox downregulated nephrin and podocin and induced apoptosis due to mitochondrial dysfunction and overproduction of reactive oxygen species (ROS) [7].

The classical biomarkers of nephrotoxicity include creatinine, urea, and uric acid [8]. Additionally, kidney injury molecule-1 (KIM-1) and neutrophil gelatinase-associated lipocalin (NGAL) are indicators of proximal and distal tubule damage in response to various types of kidney injury [9]. NGAL also linked to inflammation in several organs, including the kidney and heart [10].

Naringin (4′,5,7- trihydroxyflavanone-7-rhamnoglucoside) (NG) is a natural flavanone glycoside extracted from citrus fruits such as grapefruit and oranges [11]. NG showed remarkable therapeutic effect for treatment and prevention of various diseases and pathophysiological conditions [12]. The medicinal value of NG is mainly attributed to its antioxidant and anti-inflammatory properties. The safety and tolerance profile of NG is demonstrated by a number of preclinical settings that have been verified by toxicity studies in animal models, indicating that NG might be administered to humans without risk [13, 14].

NG showed a protective effect against nephrotoxicity caused by cisplatin, gentamicin, 5-fluorouracil and paclitaxel [15–17] as well as acrylamide-induced nephrotoxicity in rats [18]. NG demonstrated a wide range of remarkable anticancer effects in various malignancies [19, 20]. The use of flavonoids, such as NG, either alone or in combination with chemotherapy drugs may potentiate their anticancer properties. Numerous cell signaling pathways mediate its anticarcinogenic mechanisms [21]. NG has been investigated in relation to a number of cancers, such as ovarian and oral cancer [22]. NG dramatically reduced KB-1 cell viability in a dose- and time-dependent manner which may indicate a cytotoxic activity [22]. Nevertheless, there are currently no research examining effects of NG on Vero (normal kidney cells), Hep-2 (laryngeal cancer), and HCT116 (colorectal cancer). Thus, it is crucial to look into the possible anticancer activity of NG, DOX, and combination index.

Prior research has not examined the nephroprotective impact of NG on renal damage induced by DOX. Therefore, the current study aimed to reveal the potential protective impact of NG on DOX-induced nephrotoxicity and to clarify the possible biological processes and mechanism involved in its effects, with the objective of reducing kidney damage and increasing the therapeutic utility of DOX in clinical settings. Furthermore, in order to verify the anti-viability effect, this study investigated the potential anticancer activity of NG, DOX, and combination index against three different cell lines: HCT116 (colorectal cancer), Hep-2 (laryngeal cancer), and Vero (normal kidney cells), and to ruling out any potential impact on the anti-cancer efficacy of Dox. This lends credence to the current in vivo study for using NG as renoprotection during DOX treatment.

## Material and methods

### Experimental animals and design

In this investigation, adult male albino Wistar rats weighing 120 ± 5 g were obtained from VACSERA in Cairo, Egypt. The rats were then housed in the animal house of the Department of Zoology at Mansoura University. They were housed in an environment with 12-hour cycles of light and dark, a constant temperature of 24 ± 2, and regulated humidity. Standard commercial rat food and water were supplied ad libitum.

Institutional animal care and use committee (ACUC) of Mansoura University approved the legislation used in this work, which complies with international standards for laboratory animal handling and care (approval number: MU-ACUC (SC. PhD. 23.09.13)). Moreover, all procedures were conducted in accordance with the Guide for the Care and Use of Laboratory Animals endorsed by the National Institutes of Health (USA).

Following acclimatization, animals were randomly assigned into four groups, with five animals each:

Rats in the control group were given water and a typical diet without treatment.

Rats in the naringin (NG) group received an oral dosage of NG (100 mg/kg) [23] daily for 14 days. NG was provided from Sigma-Aldrich Chemicals (St. Louis, MO, USA).

Rats in the doxorubicin (DOX) group were given a single (15 mg/kg, ip) dosage of DOX [24, 25] on the 10^th^ day of the experiment. A national pharmaceutical agent in Egypt supplied a 50 mg/25 ml injectable vial of doxorubicin hydrochloride.

**Fig. 1:**
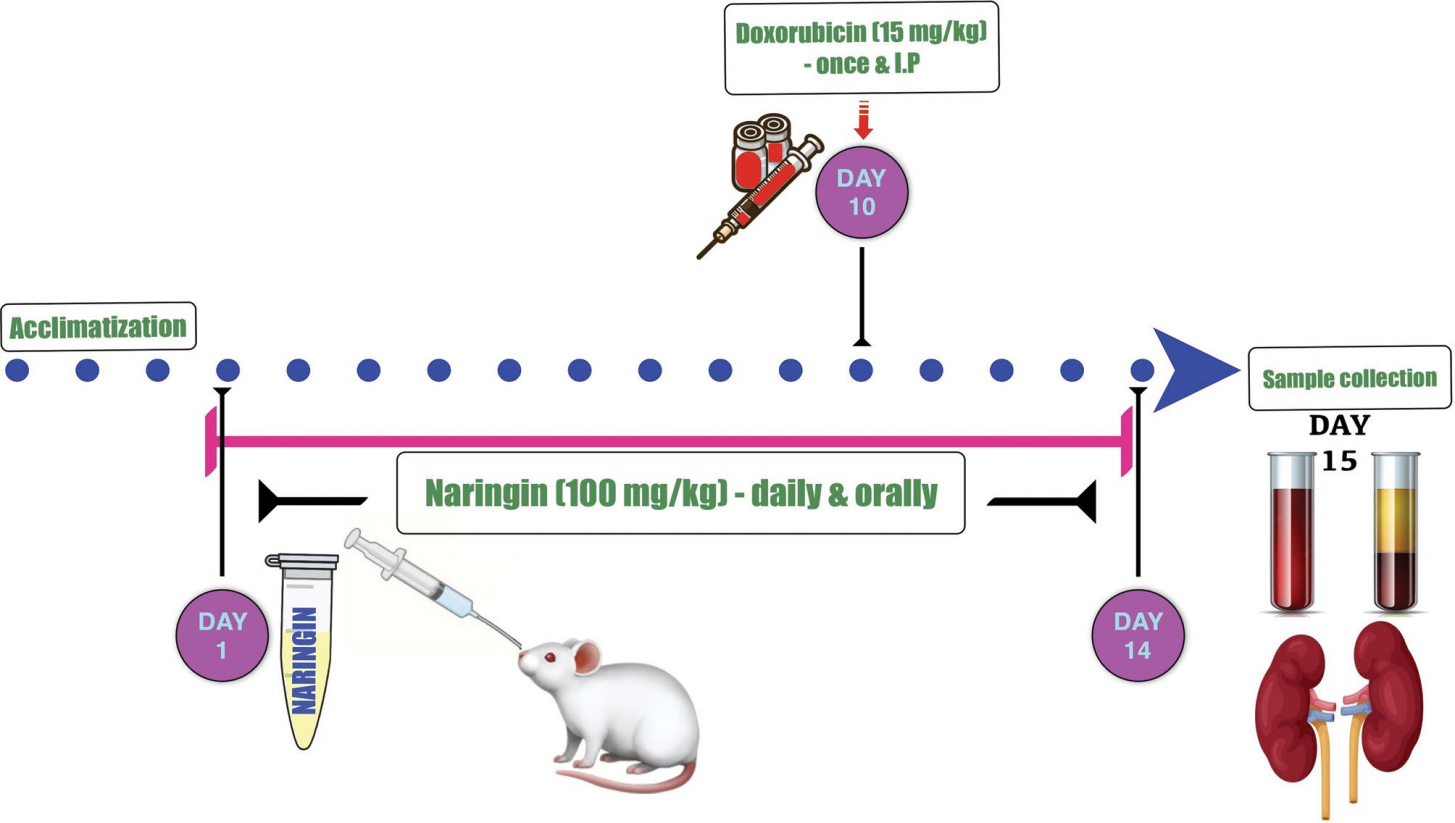
Schematic representation of the experimental design. Rats were given 100 mg/kg of naringin (NG) orally for 14 days, and on the tenth day of the examination, they received an intraperitoneal (ip) injection of doxorubicin (DOX) at a dose of 15 mg/kg. After 14 days of the experiment, overnight fasted animals were euthanized then sacrificed, followed by samples collection

Rats in the naringin + doxorubicin (NG + DOX) group received 100 mg/kg of NG orally, daily for 10 days, then were given a single (15 mg/kg, ip) dosage of DOX on the 10th day with continued daily treatment with NG for a further four days at the same dosage to evaluate the preventive role of (NG). To act as a protector, NG was given before DOX. The current experimental design and timeframe were depicted in Fig. 1.

### Samples collection and preparation

At the end of the experimental period of 14 days, rats were fasted overnight then anaesthetized with ketamine/xylazine at a dosage of 0.55 mL/100 g body weight intraperitoneally [26]. Cardiac punctures were used to withdraw blood into non-heparinized tubes then centrifuged to separate sera and stored at −20 °C for biochemical investigations. Then anesthetized rats were decapitated and both kidneys were taken out and cleaned. The right kidneys were preserved for histological analysis in a 10% neutral formalin solution. In contrast, the remaining ones were homogenized in cold Tris-HCl buffer (0.1 M, pH 7.4) and stored at −20 °C until biochemical analysis.

### Biochemical investigations in serum and kidneys

Creatinine, uric acid, blood urea nitrogen (BUN), alkaline phosphatase (ALP) activity, and lactate dehydrogenase (LDH) activity (were estimated in serum by colorimetric kits purchased from Elabscience® Biotechnology Co., Ltd., Wuhan, China.

The levels of kidney injury molecule 1 (KIM-1), neutrophil gelatinase-associated lipocalin (NGAL), nephrin, and podocin were assessed in the kidney tissue homogenate using the enzyme-linked immunosorbent assay (ELISA) kits purchased from CUSABIO (Houston, Texas, United States), following the manufacturer’s instructions.

In kidney homogenates, reactive oxygen species (ROS) (Catalog # CK-bio-20410) was estimated using ELISA kits supplied from Shanghai Coon Koon Biotech Co., Ltd, (Shanghai, China). While malondialdehyde (MDA), protein carbonyl (PC) (Catalog # EU2629), and 8-hydroxy-2’-deoxyguanosine (8-OHdG) were estimated using ELISA kits purchased from Fine Test (Wuhan Biotech, China). Furthermore, glutathione (GSH) content, glutathione peroxidase (GPx), and glutathione reductase (GR) activities in the kidney’s homogenates were estimated using My Biosource (San Diego, USA) ELISA kits following the manufacturer’s instructions. Interleukin-10 (IL-10), Interleukin-6 (IL-6), Interleukin 1 beta (IL-1β), tumor necrosis factor-alpha (TNF-α) and nuclear factor kappa B (NF-κB) levels were measured using ELISA kits supplied from CUSABIO (Texas, USA).

Total RNAs from kidney tissues were isolated and purified using RNeasy Mini Kits (Catalogue No. # 74104) (QIAGEN, Hilden, Germany) in accordance with the manufacturer’s instructions. Quantiscript reverse transcriptase was then used to reverse transcribe the RNA. Subsequently, using a Rotor-Gene Q 2plex, Qiagen qRT-PCR Cycler (Catalogue No. # 9001620), (QIAGEN, Hilden, Germany), and a QuantiFast SYBR® Green RT-PCR kit (Catalogue No. # 204156), (Qiagen, Hilden, Germany), quantitative real-time PCR (RT-qPCR) analysis was carried out in accordance with the manufacturer’s instructions [27]. The forward and reverse primers for IL-10, IL-1β, IL-6, TNF-α and, NF-κB as well as, β-actin as the internal housekeeping reference are as follows.

**Table:** 

Gene	Forward (5’ − 3’)	Reverse (5’ − 3’)
***IL-10***	CACTGCTGTCCGTTCTCC	GCTCTTCCTTCTTACCCTCA
***IL-1β***	CACCTCTCAAGCAGAGCACAG	GGGTTCCATGGTGAAGTCAAC
***IL-6***	TCCTACCCCAACTTCCAATGCTC	TTGGATGGTCTTGGTCCTTAGCC
***TNF-α***	AAATGGGCTCCCTCTCATCAGTTC	TCTGCTTGGTGGTTTGCTACGAC
***NF-κB***	GACGACACCTCTACACATAGCAG	TTCTTCTCCAGCCTTCTCCCA
**βactin**	AAGTCCCTCACCCTCCCAAAAG	AAGCAATGCTGTCACCTTCCC

The threshold cycle (Ct) values were evaluated in order to do quantitative analysis during the exponential amplification process. The fold change (ΔCt) in target gene expression was calculated using beta actin as the reference gene. The fold change in gene expression compared to the control group was calculated for each treatment group using 2 − ΔΔCt [28].

Paraffin-embedded kidney sections were deparaffinized then processed for IHC staining using the labelled streptavidin-biotin immunoperoxidase technique [29]. Briefly, sections were incubated overnight at 4 °C with primary antibodies, (anti-cleaved caspase-3 rabbit polyclonal antibody Servicebio, USA; diluted 1:500), anti-Nrf2 rabbit polyclonal antibody ABclonal, Wuhan, Hubei, China; 1:200), and anti-TGF-β1 rabbit monoclonal antibody Abcam, USA; diluted 1:500)) following the manufacturer’s instructions. Slides were then examined and photographed using an Olympus® digital camera installed on an Olympus® microscope with a 1/2 X photo adaptor, using 10 and 40X objective lenses. The resulted images were then analyzed for labeling index using Image J software as previously described.

### Histopathological and histochemical investigations

Kidney tissue samples were fixed in 10% neutral formalin, dehydrated in ethyl alcohol, cleaned in xylene, and then embedded in paraffin wax for histopathological and histochemical analysis. Haematoxylin and eosin (H&E) and Periodic Acid Schiff (PAS) were used to stain 5-μm sections. These sections were then examined and photographed using an Olympus® digital camera mounted on an Olympus® microscope with a 1/2 X photo adapter and a 10&40X objective. A special built-in procedure for glomerular and Bowman’s capsular perimeters in H&E sections and percentage of glycogenation in PAS histochemically stained sections was used to analyze the resulting pictures using image J software on an Intel® Core I7®-based computer utilizing Video Test Morphology® software (Russia).

### Cell lines and culture

Human colorectal carcinoma (HCT116), laryngeal cancer cells (Hep-2), and normal kidney cell (Vero cells) were purchased from VACSERA, Cairo, Egypt. Sigma and GIBCO (New York, USA) supplied the tissue culture reagents, which included DMEM, fetal bovine serum (FBS), penicillin/streptomycin, l-glutamine, trypsin, dimethyl sulfoxide (DMSO), and MTT. Cells were cultured in DMEM supplemented with 10% FBS and 100 unit/mL penicillin/streptomycin at 37 °C in an incubator with a 95% humidity and 5% CO2. To ensure exponential development, the cells were divided using trypsin-EDTA and passaged every two to three days.

### Cell viability assay

Cell vitality after different treatments was evaluated using the MTT test. Following a 4-hour incubation period with 5 mg/ml of MTT (Sigma), the medium was changed with 100 μl of DMSO (Sigma) and vortexed. The absorbance was measured at 570 nm. The sigmoidal curve was used to determine the concentration of DOX and NG that inhibit viability 50% of cells (IC50) using the statistical program Graph Pad (Prism).

### The combination index (CI)

The combination index (CI) was used to determine the type of interaction between the DOX medication and NG. To calculate CI, CompuSyn software using the Chou-Talalay method was used [30, 31]. A CI value greater than one indicates antagonism, CI = 1 denotes addition, and CI < 1 denotes synergism. In order to estimate CI, two cell lines were treated with NG at a concentration equivalent to its IC50 value. After two hours, DOX was added to the cells at a concentration equal to either the 3⁄4 or 1⁄2 IC50, or the cell’s IC50 value. NG was used to create each combination (n = 5/cell line) as a non-constant ratio at concentrations that matched the IC50 values by 1, 2, 3, and 4. For all treatments, cells with 60–80% confluence were employed, and they were cultivated in a CO_2_ incubator for 24 hours.

## Statistical analysis

Once the normality distribution for each group was verified, statistical analysis was conducted using Graph Pad Prism 7.02 software. The mean ± standard deviation of the mean (SD) was used to express the results (n = 5). A one-way analysis of variance and a post hoc (Tukey) test for multiple comparisons were used to assess statistical comparisons.

## Results

### NG improved kidney function in DOX-induced renal injury

**Fig. 2:**
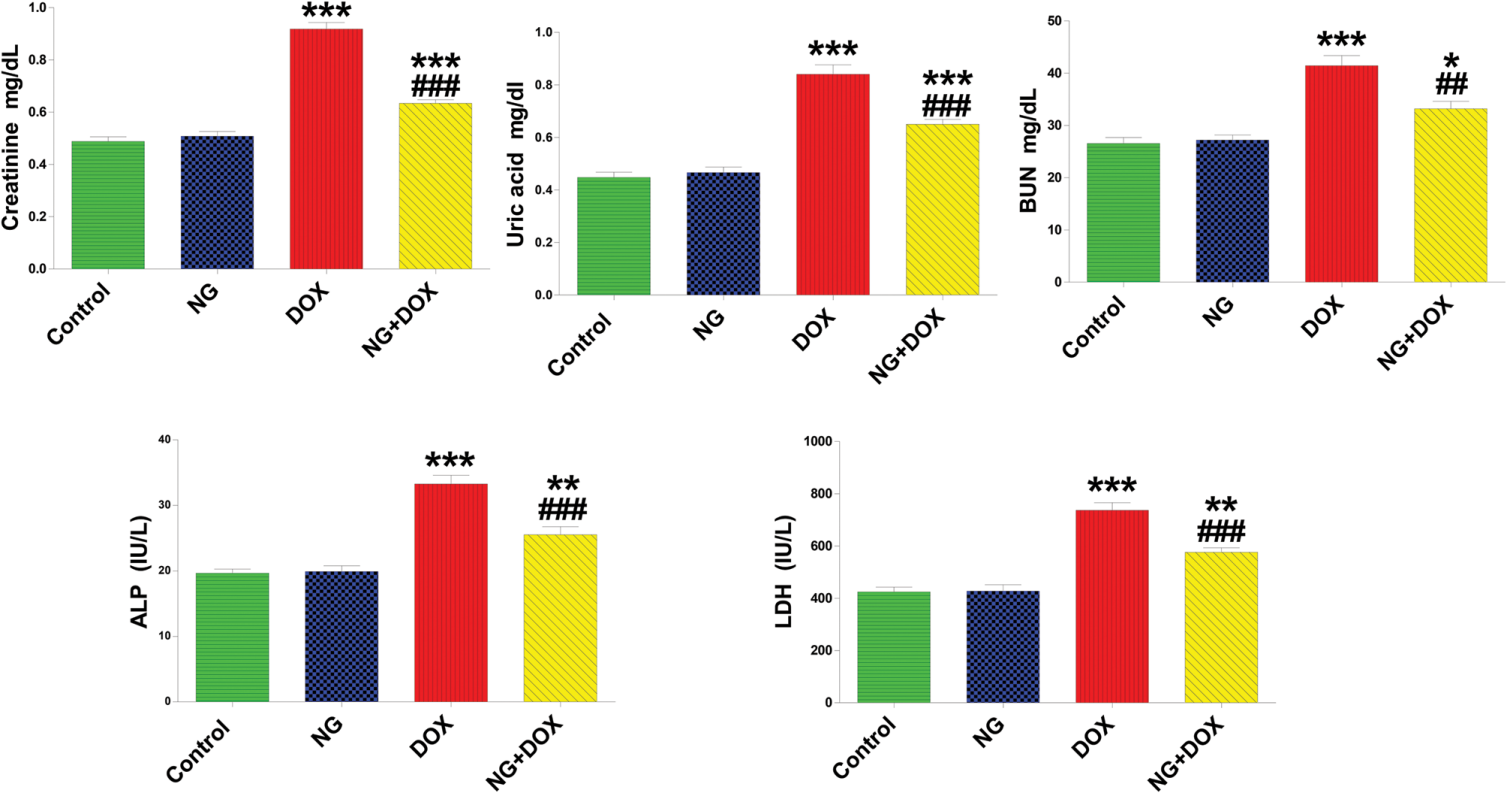
The effects of NG (100 mg/kg) and DOX (15 mg/kg) on the levels of creatinine, uric acid, blood urea nitrogen (BUN), alkaline phosphatase activity (ALP), and lactate dehydrogenase activity (LDH) in the serum of control and different treatment groups after 14 days. Values are expressed as mean ± SD (n = 5). *, ** and *** indicate statistical significance at *P < 0.05, P < 0.01 and P < 0.001*, respectively, compared to the control group. ## and ### indicate statistical significance at P < 0.01 and *P < 0.001*, respectively, compared to the DOX group

In the current study, kidney function parameters were unaffected by the daily treatment of NG for 14 days. However, as compared to the animals in the control group, a single Dox administration markedly increased the levels of creatinine, uric acid, blood urea nitrogen (BUN), and the activities of lactate dehydrogenase and alkaline phosphatase in the serum (Fig. 2) (P < 0.001). When NG was administered to rats treated with DOX, the rise in all these renal function indicators was significantly decreased.

**Fig. 3:**
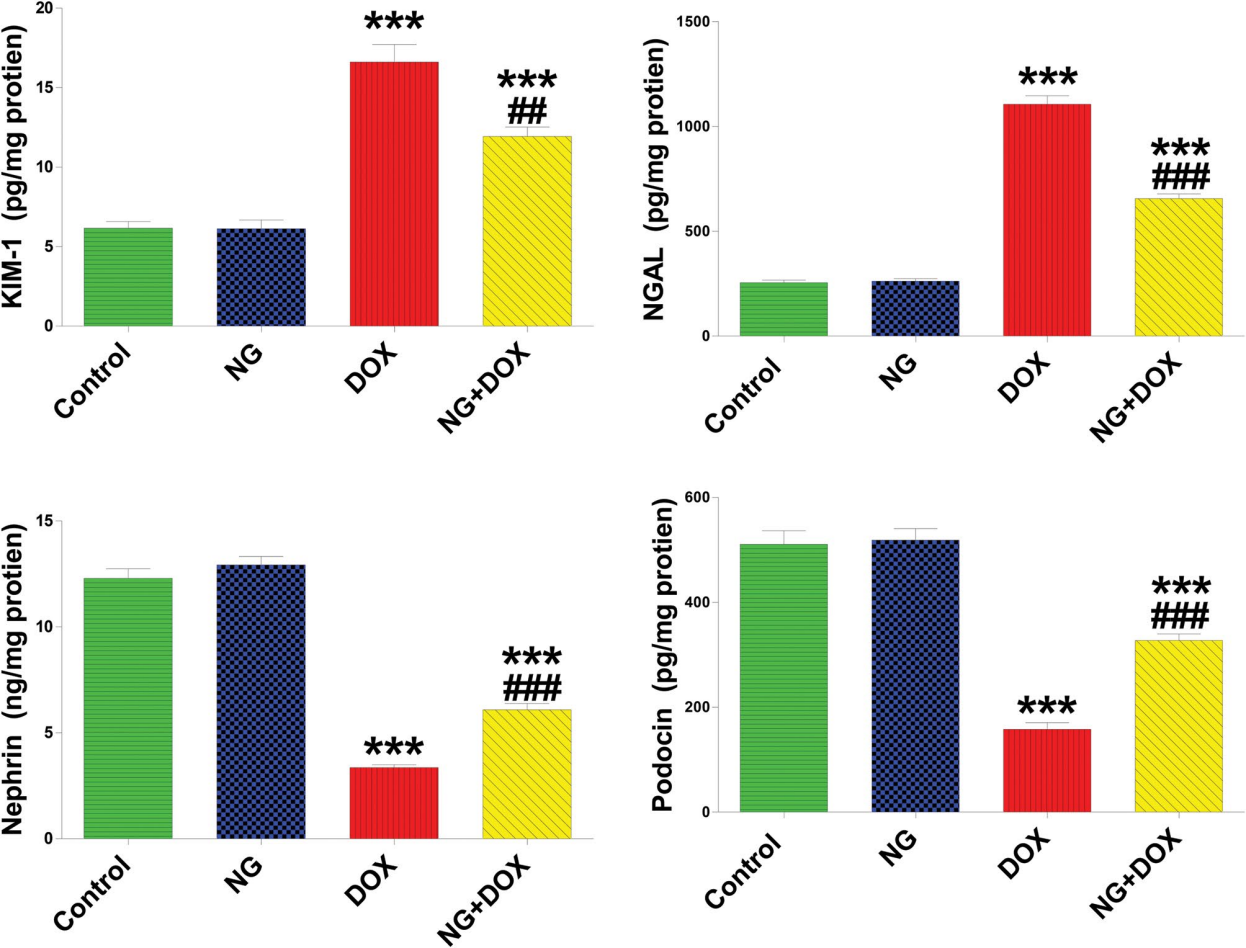
The effects of NG (100 mg/kg) and DOX (15 mg/kg) on the levels of kidney injury molecule-1 (KIM-1), neutrophil gelatinase-associated lipocalin (NGAL), nephrin, and podocin in the kidney tissues of the rats in the different groups. Values are expressed as mean ± SD (n = 5). *** indicate statistical significance at *P < 0.001*, compared to the control group. ## and ### indicate statistical significance at *P < 0.01 and P < 0.001*, respectively, compared to the DOX group

Rats given DOX showed statistically significant (P < 0.001) increases in the levels of Kim-1 and NGAL in the kidney in comparison to the control group. Conversely, NG treatment caused a significant (p < 0.01) reduction in these parameters in DOX-treated rats as compared to the DOX group. NG on its own did not affect the levels of Kim-1 and NGAL compared with the control rats (Fig. 3).

The impact of NG and Dox on kidney nephrin and podocin levels is also shown in Fig. 3. The data showed that the levels of nephrin and podocin were significantly (P < 0.001) reduced after receiving Dox therapy; this effect was significantly mitigated when NG was given prior to DOX treatment.

### NG prevented Dox-induced renal histopathology and improved polysaccharides content

**Fig. 4:**
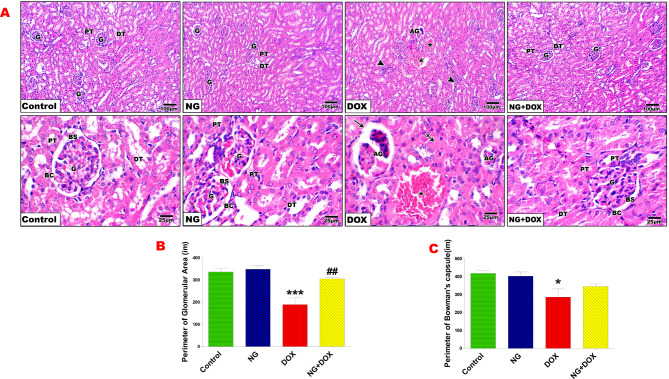
Photomicrographs of rat kidney sections from the control and different treatment groups (**A**). Control and NG groups revealing normal histological architecture represented by normal glomeruli (G), Bowman’s capsules (BC), and Bowman’s space (BS), as well as proximal convoluted tubule (PT) and distal convoluted tubule (DT). Sections of Dox-treated rats showing glomerular atrophy (AG), large and wide capsular space (arrow), dilation and congestion (*), as well as destruction of tubular cells and tubular necrosis (crossed arrow) and leukocytic infiltration (arrow head). Sections of NG + Dox-treated rats demonstrating retained normal histology of glomeruli and tubules. (H&E stain, X100&400). (**B** and **C**) Quantification is expressed as the perimeter of glomeruli and Bowman’s capsule (µm) in all studied groups, respectively. Values are expressed as the mean ± SD of 5 microscopic fields/tissue samples. * and *** indicate statistical significance at *P < 0.05* and *P < 0.001* respectively compared to the control group. ## indicate statistical significance at *P < 0.01* compared to the DOX group

Histopathological examination of kidneys in the control and NG groups showed normal renal architecture of glomeruli and tubules and their perimeter. Whereas, the kidneys of Dox-treated rats showed remarkable structural histological alterations, such as glomerular atrophy with Bowman’s capsular gap expansion, tubular dilatation, and congestion, as well as necrosis in certain regions with leukocytic infiltration. Histomorphometrically, the kidney of Dox-treated rats revealed a significant reduction in the perimeter of both glomeruli (p < 0.001) and bowman’s capsule (p < 0.05) compared with the control group. On the other hand, kidney sections from NG + Dox group revealed a remarkable amelioration of the histopathological alteration induced by Dox treatment. These NG protective effects were verified by amelioration of glomeruli and bowman’s capsule perimeter compared with that of Dox treated group (Fig. 4).

**Fig. 5:**
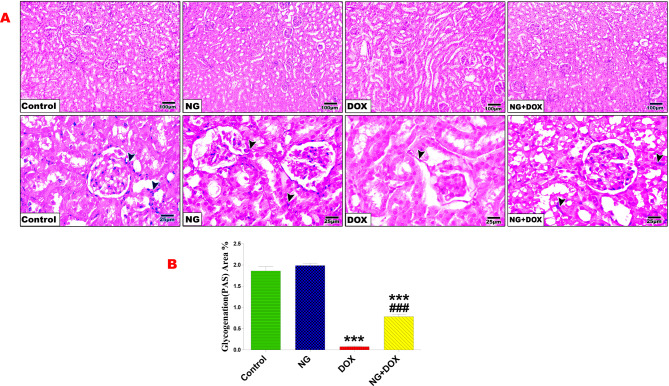
Periodic Acid Schiff (PAS) stained kidney sections of control and NG groups showing a strong positive reaction of polysaccharides in the glomerular area, Bowman’s capsule, and tubular area as well (head arrow) (**A**). Dox-treated rats revealed a significant (P < 0.001) reduction in the carbohydrate content of glomerular and many renal tubules (arrowhead). Sections of Dox + NG-treated rats exhibiting significant (P < 0.001) positive PAS reactions in glomerular and many renal tubules compared with those of the Dox-treated group (PAS X100 & 400). (**B**) Quantification of the PAS reaction in different treatment groups. Values are expressed as the means ± SD. *** indicate statistical significance at *P < 0.001* compared to the control group. ### indicate statistical significance at *P < 0.001* compared to the DOX group

Histochemical investigation of Periodic Acid Schiff (PAS) content of the kidney in the control and NG treated groups was used to stain polysaccharides in the basement membranes of the renal tubules, the Bowman’s capsule, and the glomerular tufts of capillaries. Quantification of kidney sections from all groups showed a significant (*p* < 0.001) decrease in the PAS reaction in the renal tubules and the glomeruli of the Dox-treated group compared with the control group. In contrast, a significant (*p* < 0.001) improvement in the polysaccharide content in the renal tubules and glomeruli was observed in the NG + Dox-treated group compared with the Dox-treated group (Fig. 5).

### NG improved redox balance mediators in DOX-induced renal injury

**Fig. 6:**
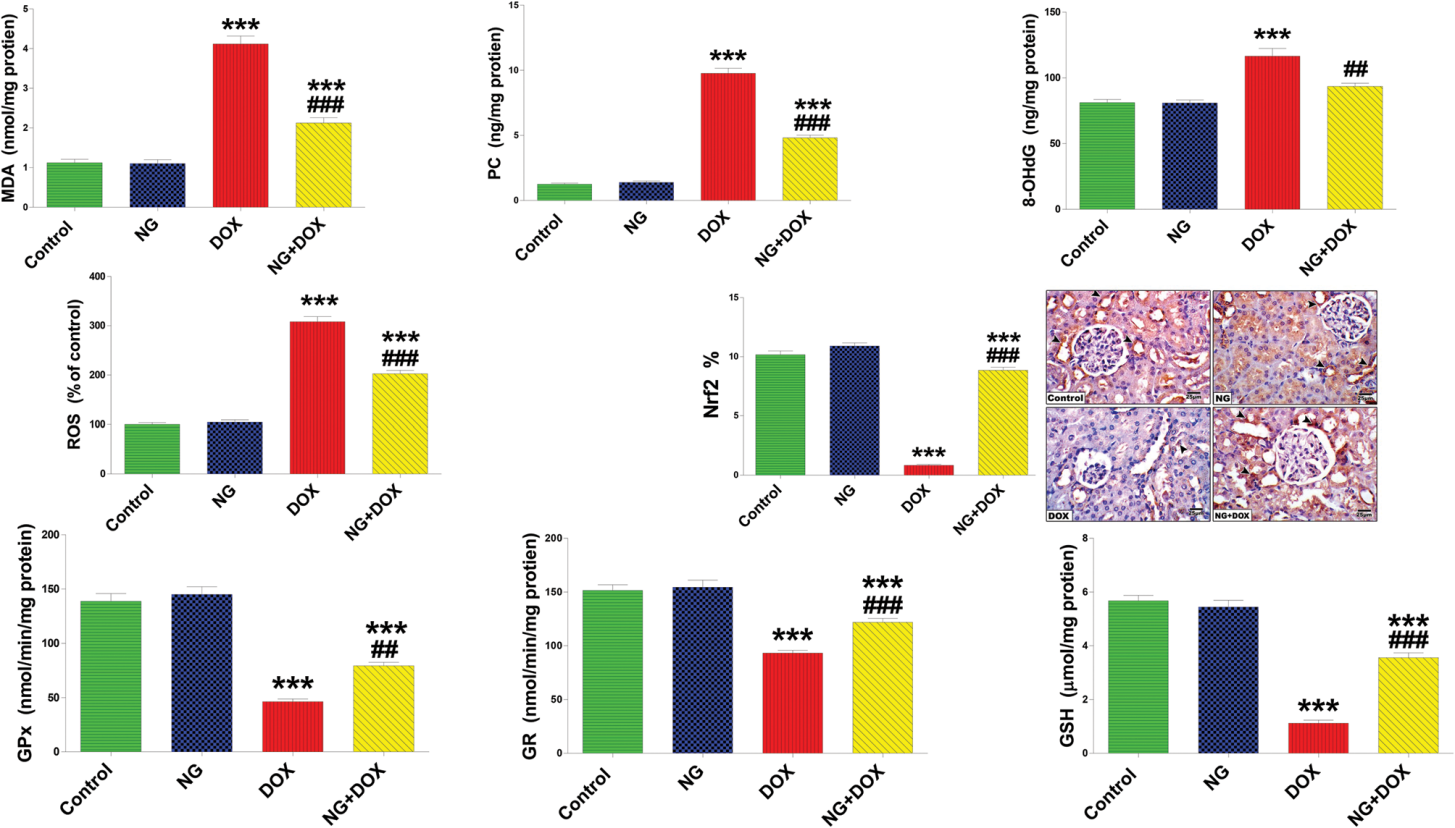
The effects of NG (100 mg/kg) and DOX (15 mg/kg) on the levels of oxidative stress biomarkers: malondialdehyde (MDA), protein carbonyl (PC), 8-hydroxy-2’-deoxyguanosine (8-OHdG), and reactive oxygen species (ROS), as well as the antioxidants, including glutathione peroxidase (GPx) and glutathione reductase (GR) activities, and glutathione (GSH) content in kidney tissues of the different treatment groups. Data are expressed as mean ± SD (n = 5). Immunohistochemical expression of Nrf2 levels in the kidney sections of control and different animal groups. Control and NG groups displayed a strong expression of Nrf2 (arrowhead). Dox-treated animals showed weak Nrf2 immunoexpression (arrowhead). Dox + NG-treated rats revealed a marked Nrf2 expression to almost normal (arrowhead) (IHC, X400). Values are expressed as the means ± SD of 5 microscopic fields/tissue samples of Nrf2 immunoexpression. *** indicate statistical significance at *P < 0.001*, compared to the control group. ## and ### indicate statistical significance at *P < 0.01* and *P < 0.001*, respectively, compared to the DOX group

Because redox balance is an important factor underpinning DOX-induced kidney injury, the ameliorative effect of NG on DOX-induced oxidative damage was explored. According to data presented in Fig. 6, there was overproduction (P < 0.001) of ROS and a significant increase in the levels of MDA, PC and 8-OHdG in the kidneys of DOX-treated rats. Additionally, the levels of GSH and the activities of GPX and GR were severely decreased (P < 0.001). To support these results, we immunohistochemically assessed the expression level of Nrf2, which illustrated a significant downregulation (P < 0.001) of Nrf2 expression in the kidneys of DOX-treated rats (Fig. 6). On the other hand, animals treated with NG before receiving DOX showed a significant improvement (P < 0.001) in oxidant and antioxidant mediators compared to DOX-treated rats.

**Fig. 7:**
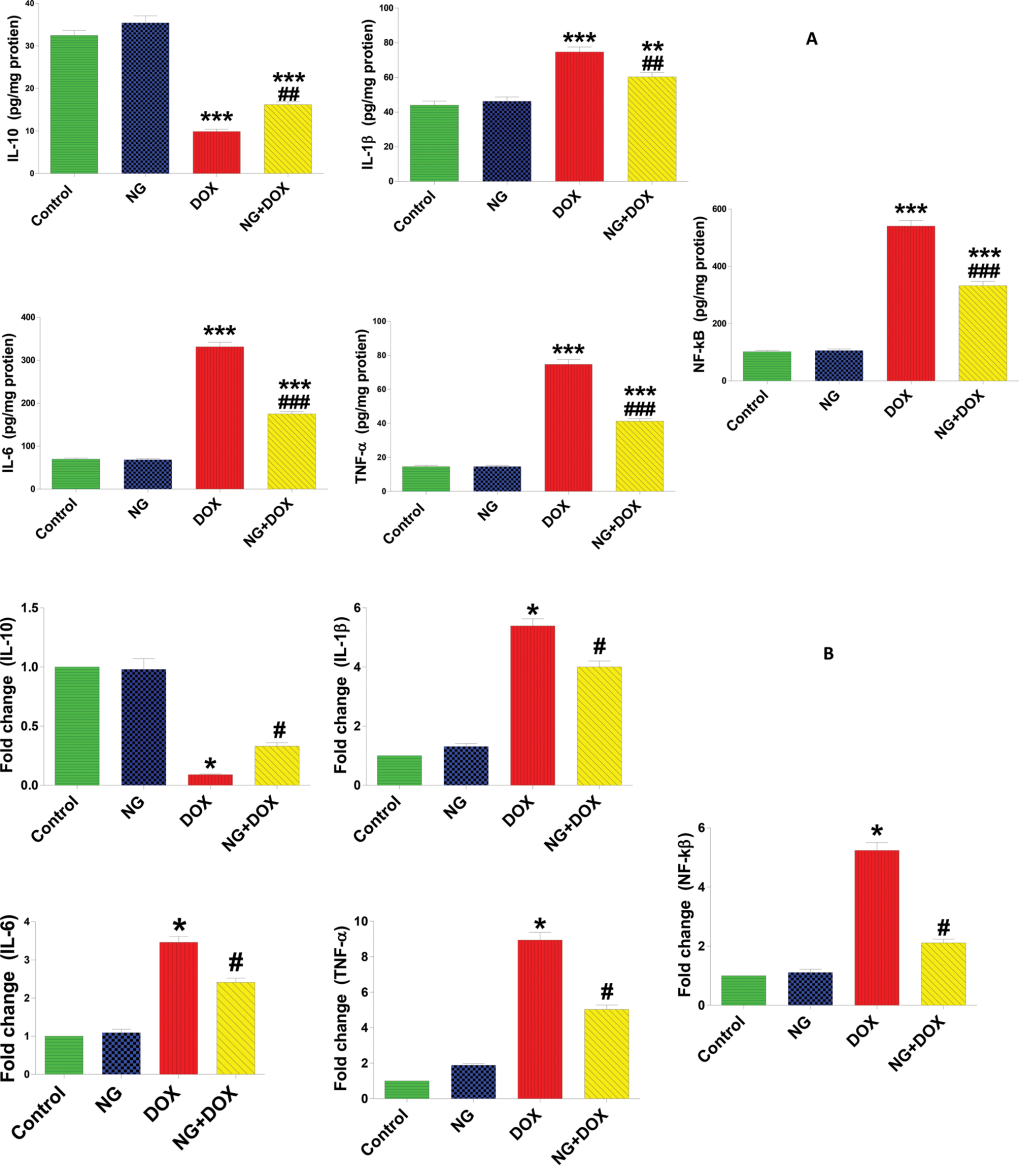
The effects of NG (100 mg/kg) and DOX (15 mg/kg) on the levels of inflammatory cytokines. anti-inflammatory; interleukin 10 (IL-10) and pro-inflammatory; interleukin 1 beta (IL-1β), interleukin 6 (IL-6), Tumor necrosis factor alpha (TNFα), and nuclear factor kappa B (NF-κB) in the kidneys of the rats in the different groups. Values are expressed as mean ± SD (n = 5). Figure 7 B. RT-qPCR analysis of (IL-10), (IL-1β), (IL-6), (TNFα), and (NF–kB) in relative gene expression kidney tissues in the different groups. Results are expressed as mean (fold change) ± SD (n = 5). ** and *** indicate statistical significance at *P* < 0.01 and *P* < 0.001, respectively, compared to the control group. ## and ### indicate statistical significance at P < 0.01 and P < 0.001, respectively, compared to the DOX group

To investigate the impact of NG on Dox-induced alterations in the levels of inflammatory mediators, the current investigation used ELIZA to measure the levels of inflammatory cytokines and RT-qPCR to detect changes in their gene expression. The data showed that Dox treatment significantly elevated the levels and gene expressions of the inflammatory cytokines (IL-6, IL-1β, TNF-α, and NF–kB) while causing a remarkable decrease in the IL-10 in tissue compared to the control animals. These changes were significantly reversed when NG was administered before DOX compared to nephrotoxic rats. (Fig. 7 A and B).

### NG decreased TGF-β1 and caspase-3 in the kidney of rats treated with DOX

**Fig. 8:**
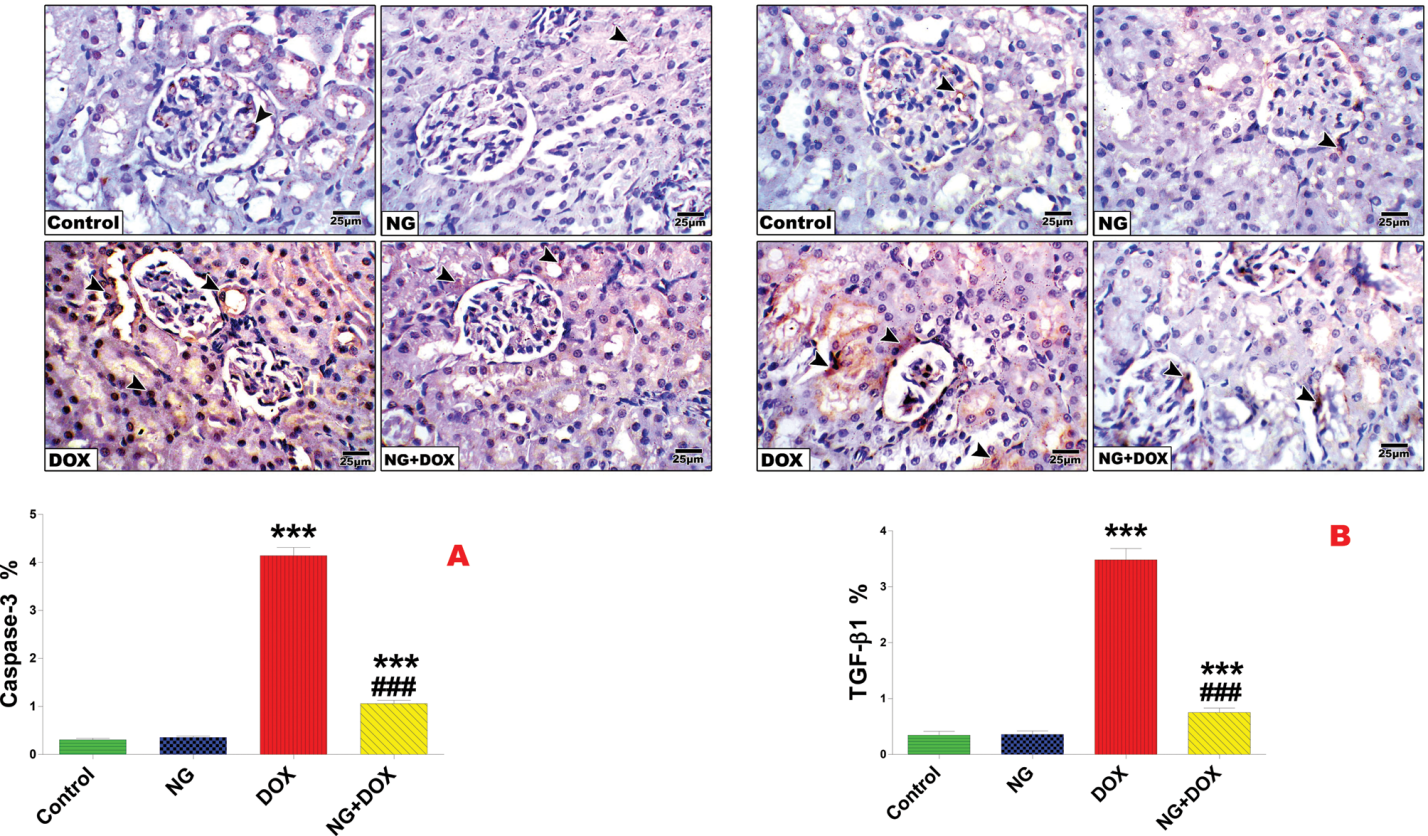
Immunohistochemical determination of the expression of caspase-3 and TGF-β1 in the kidney sections of control and different animal groups (**A**). Expression indicated by arrowhead (IHC, X400). (**B**) Values are expressed as the means ± SD of 5 microscopic fields/tissue samples of caspase-3 and TGF-β1 immunoexpression. ###, ***Very highly significant at *P* < 0.001. *** Significant as compared with the control group. ### Significant as compared with the Dox-treated group. *** indicate statistical significance at *P* < 0.001, compared to the control group. ### indicate statistical significance at *P* < 0.001, compared to the DOX group

An immunohistochemical analysis of the Dox-treated rats using caspase-3 active antibodies showed excessive (*P* < 0.001) positive staining in the glomerular endothelium and damaged tubular epithelium in comparison to the control rats (Fig. 8A). In contrast to DOX-nephrotoxic animals, immunohistochemical analysis of NG + DOX-treated rats revealed relatively few (P < 0.001) positive cytoplasmic granules in glomerular endothelium and epithelial cells and slight positivity in the cytoplasm of the tubular epithelium (Fig. 8A). The control and NG groups showed weak caspase-3 immunoexpression in immunohistochemical examination.

While some renal epithelial cells and some infiltrating cells in the interstitial of the DOX-treated rats showed strong (*P* < 0.001) intracellular staining with anti-TGF-β1 antibody, anti-TGF-β1 positive cells were not identified in the control rats. Rats given NG therapy prior to DOX injection had remarkably fewer (*P* < 0.001) positive TGF-β1 cells than rats given DOX injection (Fig. 8B).

### NG exhibited cytotoxic effects selectively on HCT116 and Hep-2, but not on Vero cells

**Fig. 9:**
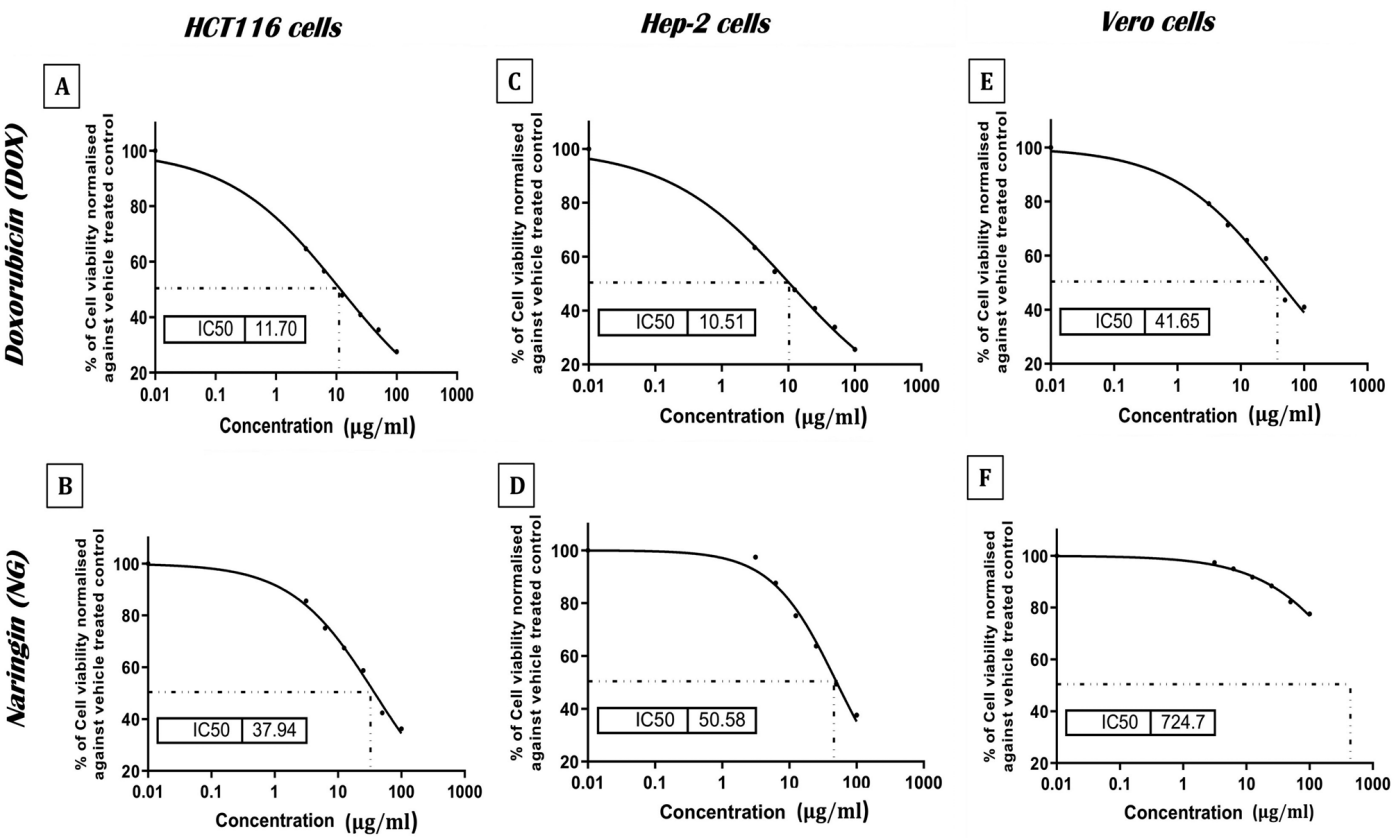
Sigmoidal curve for MTT assay showing IC50 values and the inhibition % of DOX and NG on HCT116, Hep-2 and Vero cells after 24 hrs treatment. Each data point represents an average of three independent experiments (*n* = 5)

The current investigation revealed that, the anticancer drug DOX and the natural flavonoid NG decreased the cell viability of HCT116, Hep-2, and Vero cell lines in vitro (Fig. 9). HCT116, Hep-2, and Vero viability were all suppressed by doxorubicin alone, with IC50 values of 11.70, 10.50, and 41.65 μg/mL, respectively. On the other hand, NG reduced viability with IC50 values of 37.49, 50.58, and 724.7 μg/mL, respectively. NG exhibits cytotoxic effects selectively on cancer cells (HCT116 and Hep-2), but not on normal kidney cells (Vero cell).

The non-constant combination, percentage of cell death, and combination index (CI) for NG and DOX after a 24-hour treatment are displayed in Table 1. We examined the anti-cancer effects of the DOX and NG combination on Hep-2 larynx cancer cells and HCT116 colon cancer cells, and we determined which drug combination had the strongest anti-cancer effects. Our findings showed that a combination of DOX-NG produced remarkable dose-dependent cell death on HCT116 and Hep-2. Two combinations with the lowest DOX dosages were found among the 12 combinations examined for each cell line (Table 1).

**Table 1:** Data for non-constant combination, percentage of cell death, and combination index (CI) for NG and DOX following a 24-hour treatment

IC50 ratio (DOX:NG)	HCT116			Hep-2		
	Dose(µg/ml)	Death %	CI	Dose(µg/ml)	Death %	CI
1: 1*	11.70:37.94	69.38	0.45*	10.15:50.58	72.42	0.29*
1: 2	11.70:75.88	72.91	0.55	10.15:101.16	75.15	0.36
1: 3	11.70:113.82	75.21	0.64	10.15:151.74	77.97	0.38
1: 4	11.70:151.76	81.39	0.47	10.15:202.32	82.26	0.30
¾: 1*	8.77:37.94	61.78	0.76*	7.61:50.58	67.84	0.39*
¾: 2	8.77:75.88	65.14	0.97	7.61:101.16	71.73	0.46
¾: 3	8.77:113.82	67.49	1.15	7.61:151.74	72.90	0.59
¾: 4	8.77:151.76	70.20	1.21	7.61:202.32	73.10	0.74
½: 1	5.85:37.94	50.93	1.52	5.07:50.58	54.02	1.02
½: 2	5.85:75.88	53.80	2.0	5.07:101.16	60.38	1.07
½: 3	5.85:113.83	57.91	2.1	5.07:151.74	65.72	1.01
½: 4	5.85:151.76	62.07	2.1	5.07:202.32	67.54	1.13

*The optimal CI and cell death percentage for the non-constant combination ratio that was selected for cell and molecular analysis. CI < 1 synergistic, CI > 1 antagonistic, and CI = 1 additive

## Discussion

Chemotherapy-induced nephrotoxicity is a global serious health issue that has to be addressed to increase safety margin and therapeutic utility of anticancer drugs [32]. Doxorubicin (DOX) is cytotoxic antibiotic derived from anthracycline, and has demonstrated a successful therapeutic effect on a variety of cancer types [33]. However, its clinical usefulness is limited since it damages the glomeruli and renal tubules [34]. The management of nephrotoxicity is still a major research topic, despite the many attempts to identify possible adjuvant therapy candidates. NG, a nutritional flavonoid, has gained attention recently due to its pharmacological and biological effects, which include anti-inflammatory and antioxidant properties [19, 35]. Therefore, the current study aimed to investigate the ameliorating effect of NG and possible mechanisms involved in preventing Dox-induced nephrotoxicity. There has been no study conducted so far on NG’s ability to prevent nephrotoxicity caused by DOX. The present findings showed that NG protected against Dox-induced nephrotoxicity by maintaining glomeruli and renal tubules integrity and function through its antioxidant, anti-inflammatory, and anti-apoptotic properties.

The glomeruli and renal tubules in kidney are essential for animal survival because they eliminate toxic wastes and prevent protein loss. Serum creatinine, urea and uric acid levels are critical in determining kidney function. In this investigation, the treatment with DOX caused a considerable rise in blood creatinine, urea, and uric acid levels as well as LDH and ALP activity, which was significantly mitigated by NG treatment suggesting adequate glomerular filtration rates and indicating nephroprotective effect of NG. These findings matched a recent report that NG ameliorated nephrotoxicity of various anticancer drugs [36, 37].

In the present study, nephrotoxicity from DOX was also demonstrated by substantial increases in KIM-1 and NGAL levels. Both proteins are essential components of glomeruli and renal tubule function [38] and conceded tubular injury markers [39]. Prior research on renal protective impact of NG against DOX-induced renal tubular injury was lacking. In the present study, KIM-1 and NGAL levels in kidney were markedly ameliorated by NG treatment of DOX-treated rats. These findings are consistent with the significant improvements observed in the tubular necrosis, histological tubular injury and decreased leucocyte infiltration caused by DOX treatment. After NG administration, histopathological findings and lesion scores, represented by leukocyte infiltration, in the kidneys and heart were markedly decreased by naringin [17] which supported by the current results suggesting nephroprotective effect against DOX toxicity. The amelioration of the ALP and LDH activity by NG is additional confirmation of its nephroprotective effect. The decrease in the levels of NGAL might be attributed to protective effect of NG on glomerulus and improved renal filtration. Previous experimental investigations have revealed the renal-protective benefits of NG against a variety of detrimental kidney injury conditions, including unilateral nephrectomy and I/R [40]. and Lesch-Nyhan disease in rat model [41]. Naringenin, the aglycone form of NG, showed nephroprotective effect for diabetic kidney diseases [42]. Thus, NG might exert nephroprotective effects by modulating the altered structure and function of the glomerulus and renal tubules induced by DOX.

Since nephrin and podocin are biomarkers for assessing podocyte function and podocytes are crucial to the function of glomerular filtration [43], their levels in the kidney were evaluated. In the current study, treatment of DOX resulted in a considerable decrease in nephrin and podocin levels in the kidney, indicating podocyte injury. It has been established that podocyte damage and loss are significant indicators in the etiology of glomerular injury under various conditions [44, 45]. These changes might explain the increase of creatinine, urea, KIM-1 and NGAL confirming that DOX severely damages both the renal tubules and glomeruli in the kidney [34]. On the other hand, rats treated with NG before DOX showed a considerable improvement in podocin and nephrin levels, signifying that the detrimental effect of DOX on podocytes was mitigated. Since podocyte-related proteins (nephrin and podocin) are important for the proper function of slit diaphragm, it implies that NG might maintain podocyte density and number as well as the structural integrity of slit diaphragm. These findings were supported by attenuation of glomerular disruption and suppression of enhanced vacuolar degeneration and improvement of decreased PAS-positive areas in DOX-treated rats that were protected with NG.

Antioxidants represent a vital protective mechanism in the body because of their roles in protecting cellular components from oxidative damage induced by ROS [46]. The understanding of the mechanisms by which DOX produces glomerular toxicity is still limited. But previous investigations have demonstrated that ROS are the main cause of DOX-induced nephrotoxicity [47]. DOX causes tubular interstitial inflammation and fibrosis by oxidative damage to the glomerular base membrane, podocytes, and glomerular endothelial cells [48] which the present histopathological study confirmed. In the current study, DOX generated excessive ROS, which in turn increased the oxidation of macromolecules as observed by the elevated levels of MDA, PC, and 8-OHdG in kidney tissue. Oxidative stress is assessed by biomarkers including PC, MDA, and 8-OHdG, which measure the degree of oxidation of proteins, lipids, and DNA, respectively [49]. These results, along with a drop in GSH levels and a decrease in GPx and GR activity, strongly suggested the onset of oxidative damage in the kidney. On the other hand, endogenous antioxidants include GSH, GR and GPX are defense system to maintain redox homeostasis increased ability of the kidney to scavenge toxic H_2_O_2_ and lipid peroxides [3]. This is consistent with other studies that oxidative stress is responsible for the development of proteinuria, glomerular sclerosis, and podocyte injury [34, 50]. Remarkably, NG repressed these alterations and restored redox balance and preserved histological structure of the kidney, which is in line with other earlier studies [51]. It was reported that NG supplementation remarkably increased antioxidant levels, and suppressed ROS generation and peroxidation of biomolecules suggesting the antioxidant ability of NG [52].

To explain the antioxidant effect of NG, Nrf2 expression in kidney was estimated. It is well known that the transcription factor Nrf2 controls the expression of numerous cytoprotective genes that encode antioxidant proteins, which prevents oxidative stress by triggering the production of antioxidant enzymes in response to oxidative stress thereby eliminate ROS [53]. The current immunohistochemical results showed that DOX-treated rats had significantly reduced renal Nrf2 expression in the kidney tissue. Noteworthy, NG treatment was very successful in preventing the decrease in Nrf2 expression in DOX-treated animals. In the meantime, the NF-κB level displayed the opposite trend. Therefore, it is suggested that by blocking NF-κB expression, Nrf2 activation may increase the expression of antioxidant enzymes and limit oxidative injury and inflammatory response in the kidneys of DOX-treated rats. An earlier study showed that NG prevented kidney damage by inhibiting the oxidative stress-induced NF-κB overexpression in diabetic rats by eliminating ROS, the mediator that triggered oxidative damage cascade [54]. Nrf2 has been demonstrated to have protective effects in a number of in vivo and in vitro experimental models of acute kidney injury, making it a prospective therapeutic target in chronic kidney disease [53]. According to these findings, it is suggested that Nrf2 is a master of redox regulation by which NG might exert nephroprotective and therapeutic impact.

Inflammation is a major factor in DOX-induced kidney damage. Numerous earlier investigations have documented that DOX causes inflammation and leads to excessive production of pro-inflammatory cytokines in kidney, including interleukin-6 (IL-6) and tumor necrosis factor-α (TNF-α) and NF-κB [34, 55]. Numerous genes linked to the onset of renal disease are regulated by the transcription factor NF-κB [56]. Through the mediation of TNF-α, IL-1β, and IL-expressions, NF-κB activation has a substantial impact on the pathophysiology of DOX-induced renal inflammation [57]. The current study demonstrated that DOX caused an elevation in NFκB, TNF-α, IL-1β and IL-6, and decrease of IL-10 in DOX-treated rats. Notably, treatment with NG reduced the upregulation of NFκB, IL-1β, TNF-α, and IL-6 levels caused by DOX and increased level of antiinflammatory cytokine, IL-10. In the present study, we used ELISA and real-time qPCR analysis to measure inflammatory markers in kidney tissue to confirm the effects of NG on inflammatory cytokines changed by DOX. The gene expression of pro-inflammatory cytokines was markedly suppresed while the anti-inflammatory cytokine was significantly upregulated in the DOX-treated rats compared to the DOX-treated rats. These results indicate that NG might have anti-inflammatory effects and could reduce the renal damage caused by DOX through inhibiting NF-κB and related proinflammatory mediators.

Furthermore, TGF-β1–induced apoptosis in podocytes is associated with upregulated Bax and caspase-3 levels and DNA fragmentation in early kidney injury [58]. TGF-β1 signaling may cause proximal tubule injury through de-differentiation, cell cycle arrest, and increased susceptibility to apoptosis [59]. Thus, inhibiting TGF-β1 can prevent DNA damage and apoptosis via downregulation of caspase-3. According to the current study, NG was able to prevent the upregulation of TGF-β1 and caspase-3 in the rat kidney caused by DOX, indicating that NG can protect the structure and function of the kidney. Meanwhile, NG stimulated Nrf2 and prevented oxidative stress in DOX-treated rats which could contribute to NG-induced suppression of TGF-β1.

Compared to monotherapy, combination therapy, which incorporates NG with existing anticancer medications, has gained attractiveness and shown more efficient outcomes than monotherapy in a variety of clinical settings [60]. The current study showed that NG and DOX individually suppressed the viability of cancer cell lines after 24 h of incubation. However, NG alone exhibits cytotoxic effects selectively on cancer cells (HCT116 and Hep-2), but not on normal kidney cells (Vero cells). Further, the combination of a series of DOX doses with NG produced marked cytotoxicity indicating synergistic source of a new and efficient anticancer combination. These results are in line with a recent study that demonstrated the synergistic inhibitory effect of DOX and NG on the migration and proliferation of MCF-7 cells [61] and HeLa cervical cancer cells [62]. The combined effect of DOX and NG successfully inhibited the production of STAT3, JAK1, Bcl-2, Survivin, and VEGF, while increasing the level of Bax [61]. NG has been shown to overcome multidrug resistance to a variety of drugs and inhibit several signal transduction pathways [63]. The molecular mechanisms underlying the anticancer effects of NG include inactivation of GSK3β, suppresses the gene and protein expressions of NF-κB and COX-2, down regulates JAK2/STAT3, suppresses proinflammatory cytokine and chemokines, and downregulates COX and iNOS [21]. Various reports have shown how NG in combination with other cancer drugs, including DOX, affect the cell signal transduction pathways for the various cancer types.

## Conclusions

**Fig. 10:**
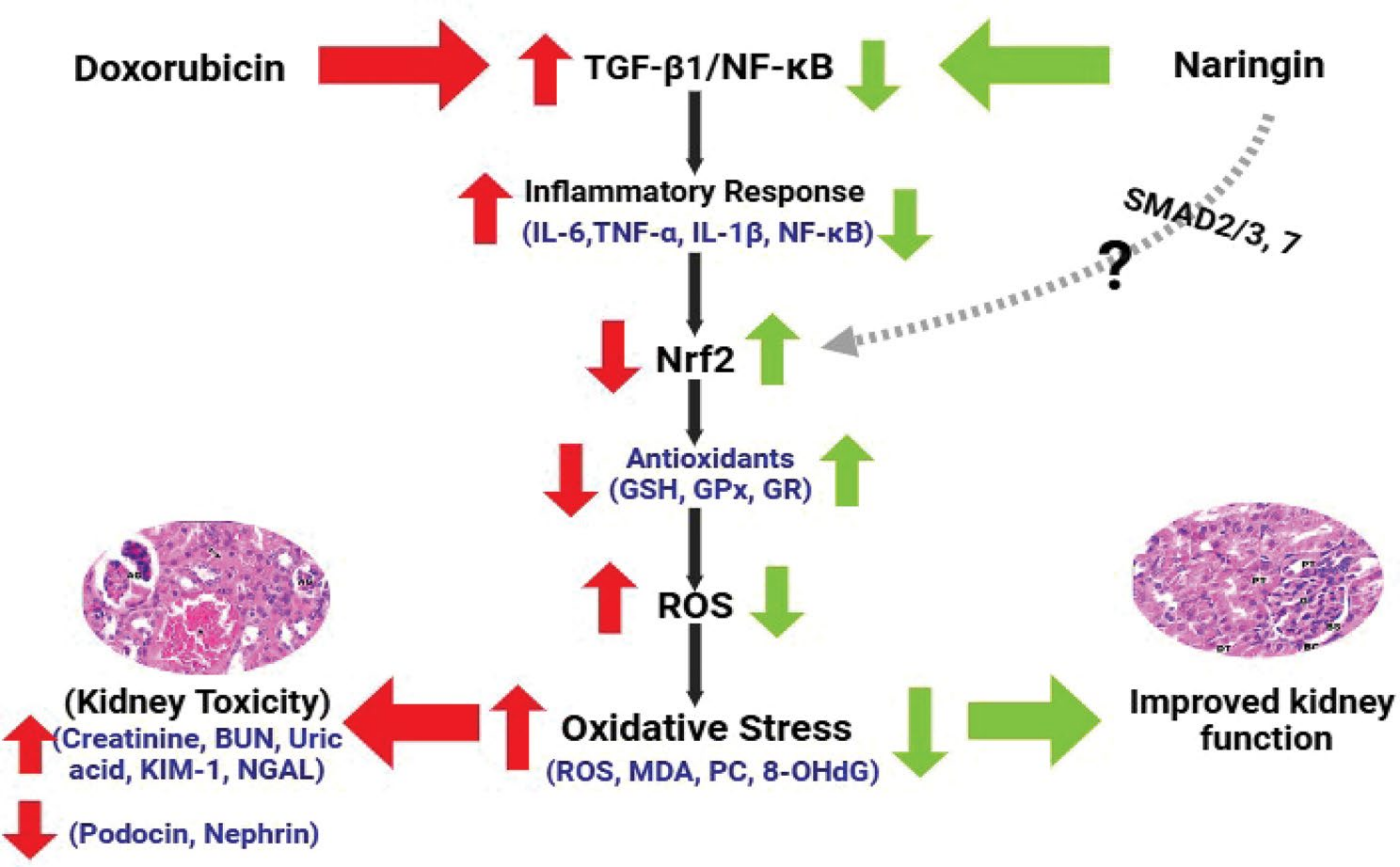
Putative mechanisms of the TGF-β1, NF-κB/Nrf2 signaling crosstalk in DOX-induced kidney injury. Under kidney lesions induced by DOX treatment, TGF-β1 release increases, most likely by podocyte and kidney cells. The activation of the TGF-β1 will, in turn, deactivate the Nrf2-dependent antioxidant response, including the inhibition of antioxidant enzymes and the development of oxidative stress. Thus, blocking the TGF-β1 receptor and its release by NG reduces this response through mechanisms that are yet to be identified (?), triggering a failure in antioxidant-related recovery

In conclusion, the positive effect of NG supplementation is attributed to the upregulation of renal Nrf2 expression and antioxidant enzyme activity as well as suppression of renal NF-κB, TGF-β1 and caspase-3 expression in kidney tissue. Moreover, NG significantly suppressed DOX-induced oxidative stress, inflammation and apoptosis, and improved the histological architecture of the kidney. Combining DOX with NG could be an effective approach to prevent the proliferation of cancer cells and mitigate DOX toxicity. Thus, NG can be used in combination with DOX to maximize therapeutic efficacy and decrease the adverse consequences of anticancer medications (Fig. 10).

## Limitations

More study of the molecular mechanisms of kidney protection in vivo is necessary to bridge the gap between laboratory results and practical application in protecting the kidney during cancer treatment, leading to a better understanding of the therapeutic potential of NG. This may include downstream signaling pathways of TGF-β1 to distinguish its pro-inflammatory, pro-fibrotic, and potential anti-inflammatory effects.

## Data Availability

No datasets were generated or analysed during the current study.
